# Experimental investigation of tidal and freshwater influence on Symbiodiniaceae abundance in *Anthopleura elegantissima*

**DOI:** 10.1371/journal.pone.0238361

**Published:** 2020-08-31

**Authors:** Daniel J. Hossfeld, Lorraine Ling, C. Sarah Cohen

**Affiliations:** 1 Estuary & Ocean Science Center, Department of Biology, San Francisco State University, San Francisco, California, United States of America; 2 Department of Genetics, Stanford University School of Medicine, Stanford, California, United States of America; University of Guam, GUAM

## Abstract

The San Francisco Bay outflow creates a tidally influenced low-salinity plume that affects adjacent coastal sites. In the study region, *Anthopleura elegantissima* (Cnidaria; Anthozoa) hosts a single symbiont, the dinoflagellate *Breviolum muscatinei*. Salinity, temperature, and aerial stress induce a bleaching response similar to corals where symbionts are expelled, causing further energetic stress. Using field observations of environmental conditions and symbiont abundance at sites on a gradient of exposure to estuarine outflow, along with a fully crossed multifactorial lab experiment, we tested for changes in symbiont abundance in response to various combinations of three stressors. Lab experiments were designed to mimic short term outflow events with low salinity, high temperature, and aerial exposure treatments. The lab aerial exposure treatment was a statistically significant factor in suppressing symbiont repopulation (ANOVA, p = .017). In the field, symbiont density decreased with increasing tidal height at the site closest to freshwater outflow (ANOVA, p = .007), suggesting that aerial exposure may affect symbiont density more than sea surface temperature and salinity. Unanticipated documentation of survival in 9 months of sand burial and subsequent repopulation of symbionts is reported as a six-month extension to past observations, exemplifying strong tolerance to environmental insult in this Cnidarian mutualism. The study of this symbiosis is useful in examining predicted changes in ocean conditions in tidepool communities and considering relative sources of stress.

## Introduction

Marine organisms living in the rocky intertidal zone are hardy to multiple stressors, but a changing climate is altering life even for many robust species. The rocky intertidal zone is an accessible marine habitat with a history of studies comparing population dynamics to changing ocean conditions [1–4]. Recent studies show climate change impacts on communities of intertidal marine invertebrates, especially considering cascading effects from altered temperature regimes in both water and air [5–12]. Zonation within the intertidal is described by regions across vertical height ranges that are stratified due to a combination of processes including aerial exposure at low tide, biotic interactions, geologic features, and other environmental variables [8,9]. Organisms at higher vertical zones experience more exposure which can alter body temperature, ambient temperature, desiccation time or salinity in isolated pools. In tidepools, coastal freshwater input and precipitation can also directly affect the salinity and can alter organism behavior [13,14].

### Environmental change in the intertidal zone

Continuing global-scale changes in rainfall patterns and increasing intensity of storm events are expected as anthropogenically-amplified climate change progresses [15–17]. While uncertainties exist in projections of future rain and snowfall decreases in California [18], storm events are expected to increase in intensity [19–21]. Average aerial temperatures, annual extreme heat days, and sea surface temperature are expected to increase in California [18,22].

The San Francisco Bay is the meeting point of the Pacific Ocean with a watershed covering 40% of California’s land. Freshwater outflow can create a low-salinity plume outside of the Golden Gate, exacerbated by a receding tide and freshwater runoff events. Predictions of extreme rainfall suggest more low salinity events at the Golden Gate in the future [19]. The effects of this plume on local organisms are not well studied, although freshwater is a well-known stressor on saltwater organisms [23–27]. Previous studies indicate the ecological importance of the outflow in local marine habitats for populations of *Leptasterias* sea stars [28,29]. The freshwater plume stays on the ocean surface and affects near-surface habitats including the rocky intertidal zone [30]. The plume is driven by snowmelt runoff and precipitation events from March to May [31]. Due to the bathymetry in the Bay, the northern side of the Golden Gate tends to have lower salinity than the southern side [32].

### Study system

The aggregating anemone *Anthopleura elegantissima* (Cnidaria; Anthozoa) is a commonly observed, abundant rocky intertidal organism found on the eastern boundary of the Pacific from Alaska to Baja California. This species can reproduce asexually (clonal fission) and sexually (broadcast spawning). *Anthopleura elegantissima* maintains a symbiotic relationship with photosynthetic algae that live and reproduce within the tentacles, providing 13 to 45% of the energy in the anemone’s diet and altering its behavior [33–36]. Near San Francisco Bay, *A*. *elegantissima* hosts one thermally tolerant symbiont, *Breviolum muscatinei* (Symbiodiniaceae) [37–42]. Like many reef-building corals, *A*. *elegantissima* harbors Symbiodiniaceae algae in a nutritive symbiotic partnership. Stressful conditions including temperature and salinity extremes produce a bleaching response similar to that of corals [43,44] where the anemone expels the symbionts. Past laboratory studies indicate a relationship between environmental conditions including exposure, temperature, light and the density of symbionts present in *A*. *elegantissima* [44–46]. Expulsion of the symbiotic algae can cause further stress due to the loss of an energy source [33]. The single stressor of low-salinity has been explicitly tested in field and laboratory studies on *A*. *elegantissima* in southern California [47], showing significant (greater than 20% loss) bleaching effects when anemones were exposed to varying amounts of freshwater for five days and longer. Low salinity also induced bleaching in an anthozoan coral (Scleractinia) [48]. Intertidal anemones are also subject to fluctuations of sediment from wave action [33], and *A*. *elegantissima* have previously been observed surviving burial events as long as three months [49].

The study of stress tolerance in cnidarian-algal symbiosis is crucial to understanding implications of the ongoing widespread coral bleaching events damaging reefs worldwide. Symbiosis can potentially increase acclimation and adaptation capabilities in symbiotic species affected by climate change [50]. Many two-factor experiments have shown additive or synergistic interactions that intensify the severity of stress; heat and light are the most common co-stressors [51,52]. This study provides a temperate intertidal example of altered host-symbiont interactions under varied and multifactorial stresses related to anticipated climate change. It further provides foundational knowledge for the potential of niche construction in the symbiosis between Symbiodiniaceae and *A*. *elegantissima* [53].

While significant long-term species-level monitoring exists (Multi-Agency Rocky Intertidal Network, Partnership of Interdisciplinary Studies of Coastal Oceans), acute changes in community structure are potentially overlooked due to less frequent monitoring time scales [54]. A controlled experimental approach linked to field comparisons can quantify tolerance thresholds of *A*. *elegantissima* to low-salinity, high-temperature water parcels and provide greater understanding of the stress accompanying large runoff events. Additionally, warming aerial temperatures have the potential to drastically affect intertidal communities during low tide. Simulated exposure scenarios in a controlled lab experiment can determine the relative effect of this known stressor to temperature and salinity stress.

In this study, we tested the effects of a low-salinity plume stemming from rainy season runoff events out of the San Francisco Bay. We examined three factors of salinity, seawater temperature, and aerial temperature stress on the *A*. *elegantissima* / *B*. *muscatinei* symbiosis in field surveys and fully-crossed lab experiments. The bleaching response to this combination of stressors is untested in lab or field studies. We evaluated the hypotheses that relatively high temperature and low salinity compared to local ocean conditions force the escalated expulsion of symbionts from the tentacles of *A*. *elegantissima*, and that aerial exposure forces further expulsion.

## Materials and methods

### Field setting

**Fig 1:**
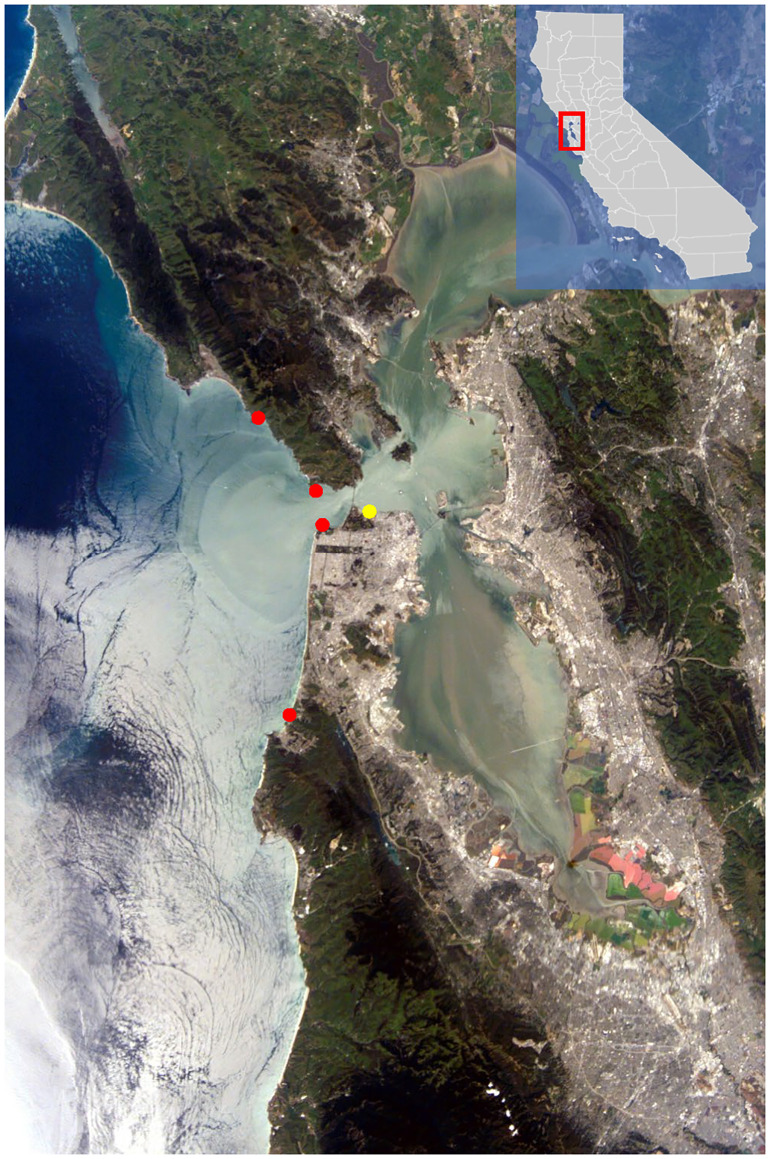
Field sites North to South: Slide Ranch, Point Bonita, Mile Rock Beach, and Rockaway Beach. Sites superimposed as red dots on a satellite image of the freshwater plume, seen as lighter color, exiting the SF Bay on 04/21/2002. The Fort Point buoy is represented with a yellow dot. White clouds cover the ocean at Rockaway Beach and further south. Image: NASA, accessed at https://eol.jsc.nasa.gov/searchphotos/photo.pl?mission=ISS004&roll=E&frame=10288.

Field surveys of *A*. *elegantissima* colonies were used to test the prevalence of bleaching in relation to estuarine output and ocean conditions. Four sites were selected along the outer coast near the San Francisco Bay outflow. The four sites north to south include Slide Ranch (14.5km north from outflow, Marin County [37.875664, -122.603617]), Point Bonita (5.6km west of the Golden Gate Bridge, Marin County [37.819426, -122.529200]), Mile Rock (4.7km west of the Golden Gate Bridge, San Francisco County [37.785931, -122.508387]), and Rockaway Beach (25km south from outflow, San Mateo County [37.607719, -122.499418]) (Fig 1). All surveys were conducted under permits obtained from the National Park Service (Permit ID GOGA-2017-SCI-0025) for Slide Ranch, Point Bonita, and Mile Rock and the California Department of Fish and Wildlife (Permit #1042178169) for all sites. *Anthopleura elegantissima* was not considered endangered or a species of concern at the time of publication. One near-outflow site in the Golden Gate Strait, Mile Rock, was selected to determine the influence of burial and low light on tidepools and was excluded from other analyses. Sea surface temperatures were measured using buoy data from Fort Point (Fig 1) (data available from Bodega Ocean Observing Node) and confirmed with a YSI 2030.

#### Colony sampling across sites

**Fig 2:**
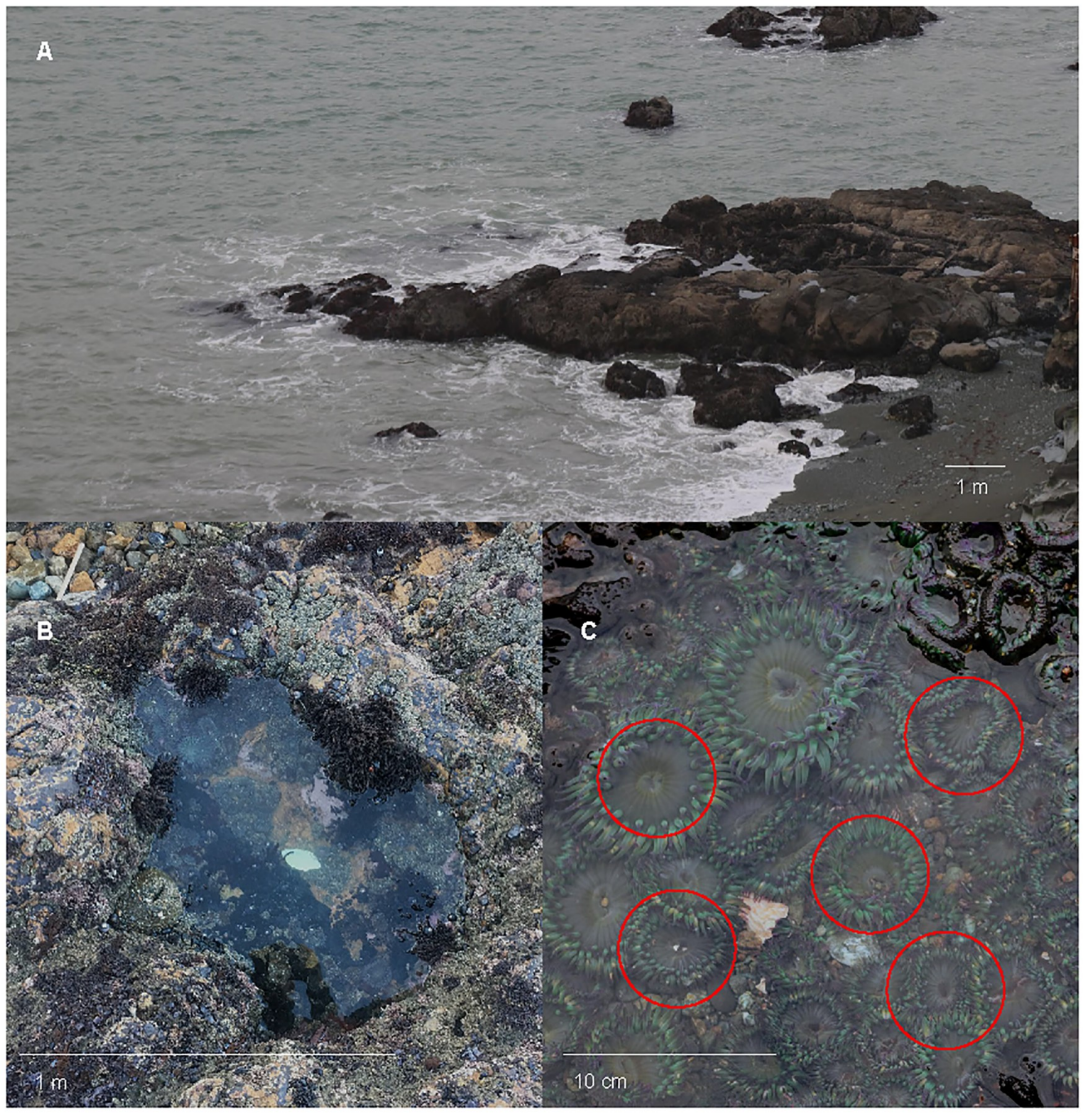
Example of sampling regime in three pools at a single site. Three pools per site were chosen based on colony presence and tidal height of each pool. Five anemones within pools were randomly selected (circled in red) and sampled for tentacles (3–4 per anemone) to assess symbiont density change over time. A) Example of vertical height change across survey pools at Point Bonita B) Pool at Point Bonita with a single colony C) Close up view of an *A*. *elegantissima* colony.

*Anthopleura elegantissima* forms colonies of genotypically identical individual polyps via asexual reproduction. To assess differences in colonies at sites with varying distance from the San Francisco Bay outflow, three to five tidepools containing a single colony were sampled at varied tidal heights at each site (Fig 2). In each colony, at least five anemones were sampled, using dissection scissors to snip and collect three tentacles per anemone. Each of the 14 total colonies across all four sites were sampled approximately monthly.

Tidal height was measured following tidepool selection using a stadia rod along with a sight level. Corrected tidal height from NOAA (available at tidesandcurrents.noaa.gov) was used to calculate vertical tidal height. All pools containing anemones were above Mean Lower Low Water (MLLW).

Colony selection criteria included polyp counts greater than 10 anemones/colony, light exposure, and accessibility. Light availability was chosen as at least 50% of daylight exposure without shading (e.g., from a nearby cliff face or nearby boulders) except for “low light” colonies. Mile Rock samples were excluded from some statistical tests due to the lack of comparable light and tidal height settings.

#### Sampling scheme within colonies

Colonies of *A*. *elegantissima* were photographed, counted, and surveyed for potential signs of bleaching including translucence and discoloration. Closed anemones in tidepools were shaded for ease in tentacle collection by manually adding algal cover, which enabled them to open. Colonies were divided into inner and outer portions which were then gridded, and anemones randomly selected into each category to avoid effects of intercolonial differences [55].

### Controlled laboratory setting

#### Animal care & feeding

Anemones were collected from the two sites north of the outflow: Slide Ranch (far from outflow) and Point Bonita (near/in outflow). Twenty anemones were collected from each site at +0.18m above MLLW. Mimicking common ocean conditions found offshore of San Francisco Bay, anemones were maintained in seawater tables ~14°C and 32ppt on a 24 hour light cycle (14 light, 10 dark) at relatively low light levels comparable to low clear daylight intensity (10μmol/m2/s measured at 0.1m depth). Prior to experiments, collected anemones were fed once per week by dropping limpets (*Lottia* spp. collected from field sites) directly into the oral disk. Shell length of limpets was measured using calipers and ranged from 4mm to 10mm. Success of feeding was evaluated the following day by looking for empty shells or live limpets. Anemones were not fed for the duration of experiments.

#### Laboratory experimental setup

After one week of acclimation under conditions described above, anemones were individually placed in separate tanks with isolated circulation and strong aeration. Then, anemones were subsequently randomly assigned to treatments shown in Table 1. Temperature was measured using Onset HOBO Pendants (Part # UA-002-64). Salinity was measured using a calibrated YSI 2030, which measures in units of parts per thousand (ppt). All salinity measurements and treatments are reported in ppt for consistency. In the Pulsed 3-Stressor Experiment, anemones were placed in submerged treatment conditions for three hours a day and were exposed to the air, simulating a low-tide event for a further three hours each day if they were assigned to the “Exposed” treatment group. “Non-exposed” anemones in the Pulsed 3-Stressor Experiment were kept in control conditions outside of the three-hour salinity and temperature stress treatment periods. The experimental duration was based on the extent of past freshwater pulses measured at the Bodega Ocean Observing Node near the outflow of the San Francisco Bay and on the earliest signals of bleaching in a past single stressor low salinity study [47]. An experimental time of five days was consistent with both factors. The timing of pulsed stressors (3 hours) during experiments was consistent with intra-daily patterns associated with tidal fluxes. Tentacles were collected on days one, three, and five.

**Table 1:** Experimental design for wet lab treatments.

Treatment	Temperature	Salinity	Air	N per treatment
	(°C)	(ppt)	Exposure (Yes/No)	
Control	14	32	N	5
1-stressor Aerial exposure (AE)	14	32	Y	
1-stressor Low salinity (LS)	14	25	N	
2-stressor LS- AE	14	25	Y	
1-stressor High temp (HT)	17	32	N	
2-stressor HT-AE	17	32	Y	
2-stressor HT-LS	17	25	N	
3-stressor HT-LS-AE	17	25	Y	

Five individual anemones were assigned to each treatment.

#### Symbiont quantification

Tentacle snips were placed in 1.5ml plastic sample tubes with artificial seawater and immediately frozen in a -20°C freezer. Samples collected in the field were put on ice in transit to the freezer. Samples were homogenized in artificial seawater with a Fisher Powergen 125 rotostat and centrifuged to separate symbiont cells from supernatant. 50μl of supernatant was used for the Modified Lowry Method to normalize symbiont count to grams of animal protein as used in past studies [43,44,56]. 25μl of the remaining symbiont homogenate was pipetted into 225μl of artificial seawater with 0.01% SDS. Samples were loaded into a Guava easyCyte HT 2-laser flow cytometer and analyzed using settings for counting Symbiodiniaceae previously used in studies with *Aiptasia* anemones and their symbionts [57]. Of the 306 samples collected in the field and laboratory experiments, 275 were viable for statistical analysis. The most common reasons for inviability included failure to sample adequate tissue and inadequate amount of protein for a reliable Modified Lowry assay.

### Statistical analysis

Symbiont density was standardized to animal protein and reported as cells/μg animal protein. Means are reported throughout the Results with accompanying Standard Deviation (SD) values. A Shapiro-Wilk Test of Normality upheld the assumption of a normal distribution among the samples. One-way Analysis of Variance (ANOVA) tested the influence of tidal height on symbiont density. In all tests, the dependent variable was symbiont number per gram animal protein. Independent variables for wet lab experiments included water temperature, salinity, and aerial exposure. For field samples, independent variables included water temperature, salinity, aerial exposure during low tide, and site. A three-way ANOVA tested the influence of salinity, temperature and air exposure on wet lab data. This test was a fully-crossed assessment of the relationship between Symbionts/Temperature, Symbionts/Salinity, Symbionts/Air Exposure, as well as the relationship of symbionts to multifactorial changes. For ANOVA, p < .05 was considered sufficient to reject the null hypothesis. Tukey’s Honestly Significant Difference (HSD) tests determined site effects and inter-treatment differences. All statistical analysis was done in R [58]. Post-hoc power analysis on ANOVA and a two-sample t test was conducted using Cohen’s conventional large effect size within the pwr package [59].

## Results

### Temperature and freshwater input

**Fig 3:**
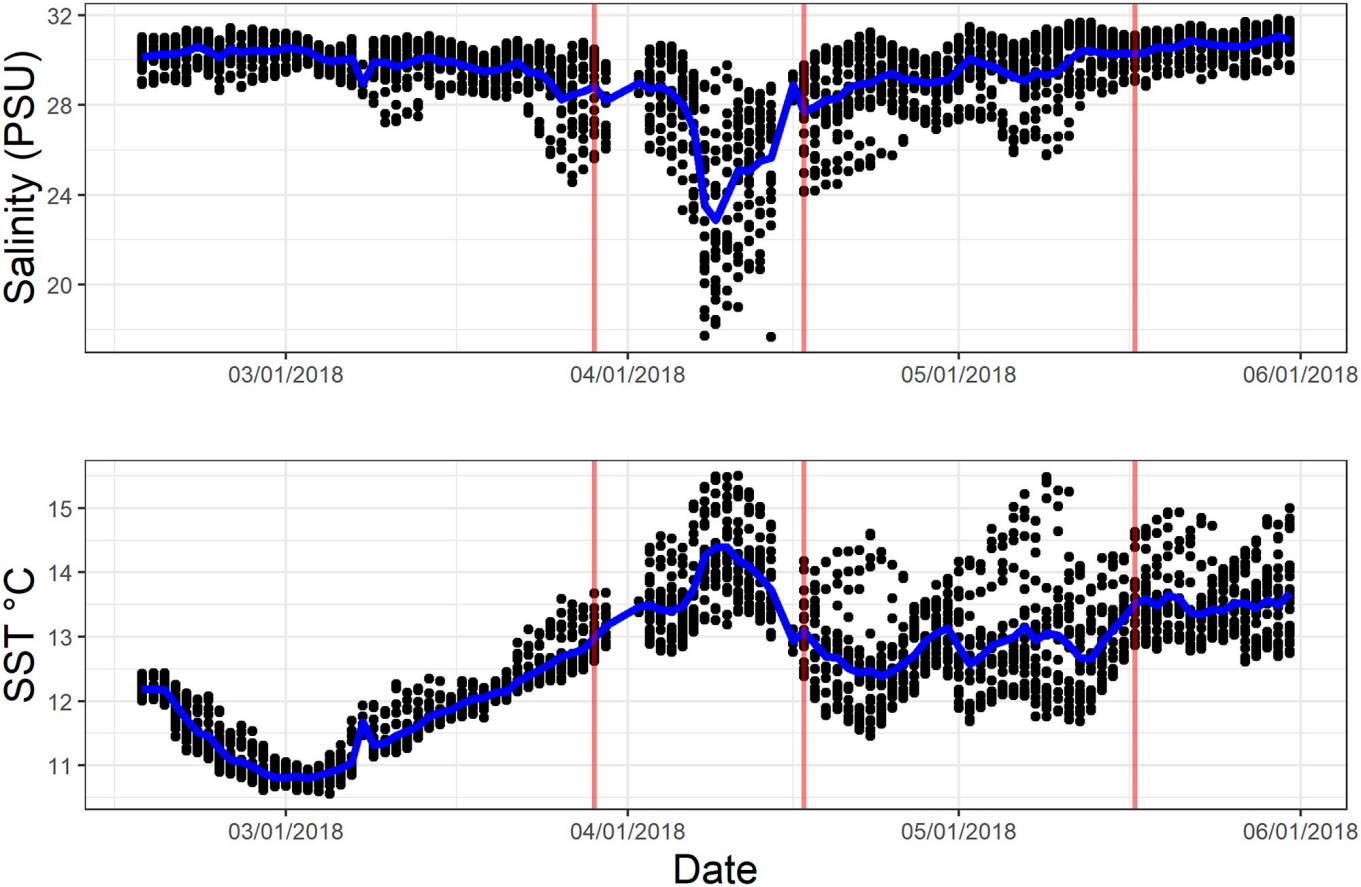
Salinity and temperature measurements at the Fort Point buoy during the survey period. Black dots show individual sampling measurements, the blue line shows daily average. Anemone sampling dates at Point Bonita are shown as red lines. One major freshwater outflow event occurred during the sampling period along with multiple minor events. Sea surface temperature (SST) was not as high as documented in past low salinity events. The most extreme low salinity period was roughly 10 days, longer than many outflow events in the past. Salinity at the Fort Point Buoy is measured and reported in PSU, equivalent to parts per thousand (ppt) reported in our experimental section.

Daily average SST ranged from 10.7°C to 15.3°C at the Fort Point buoy from 02/15/2018 to 06/01/2018. The buoy documented a decrease in average daily salinity (reported in PSU, equivalent here to ppt) during the survey period due to outflow from the San Francisco Bay and Delta (Fig 3). A 28 PSU reading translates to the presence of roughly 10% freshwater.

### Field setting

**Fig 4:**
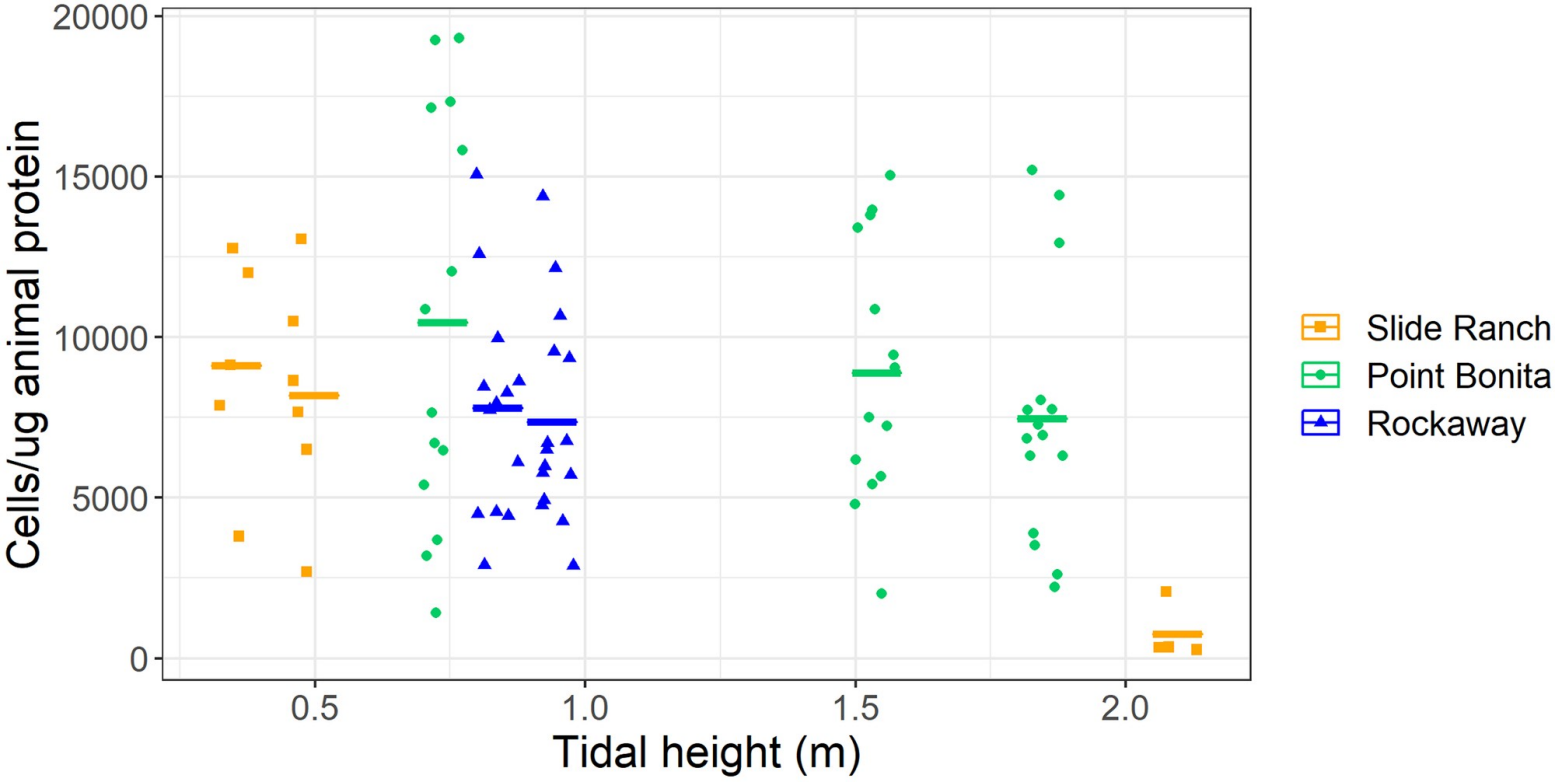
Symbiont abundance values across all surveyed pools. Individual anemones sampled for symbiont abundance are represented as dots, shown with increasing tidal height. Bars show the mean at each tidal height sampled. Mean symbiont density decreases as tidal height increases when looking at individual sites, but the pattern is less pronounced when all sites are grouped together.

Symbiont density decreased with tidal height (ANOVA, F = 4.5, p = .036) (Fig 4). The samples ranged across three sites and across tidal heights ranging 0.3m to 2m above MLLW. The highest pool from Slide Ranch appears somewhat different from the rest of the pools (Tukey HSD, p < .2 for all pools) and when the highest pool is excluded, symbiont density is no longer dependent on tidal height when looking across all sites (ANOVA, p = .42). Site-specific results including statistically significant relationships between tidal height and symbiont abundance at Point Bonita are reported below.

**Fig 5:**
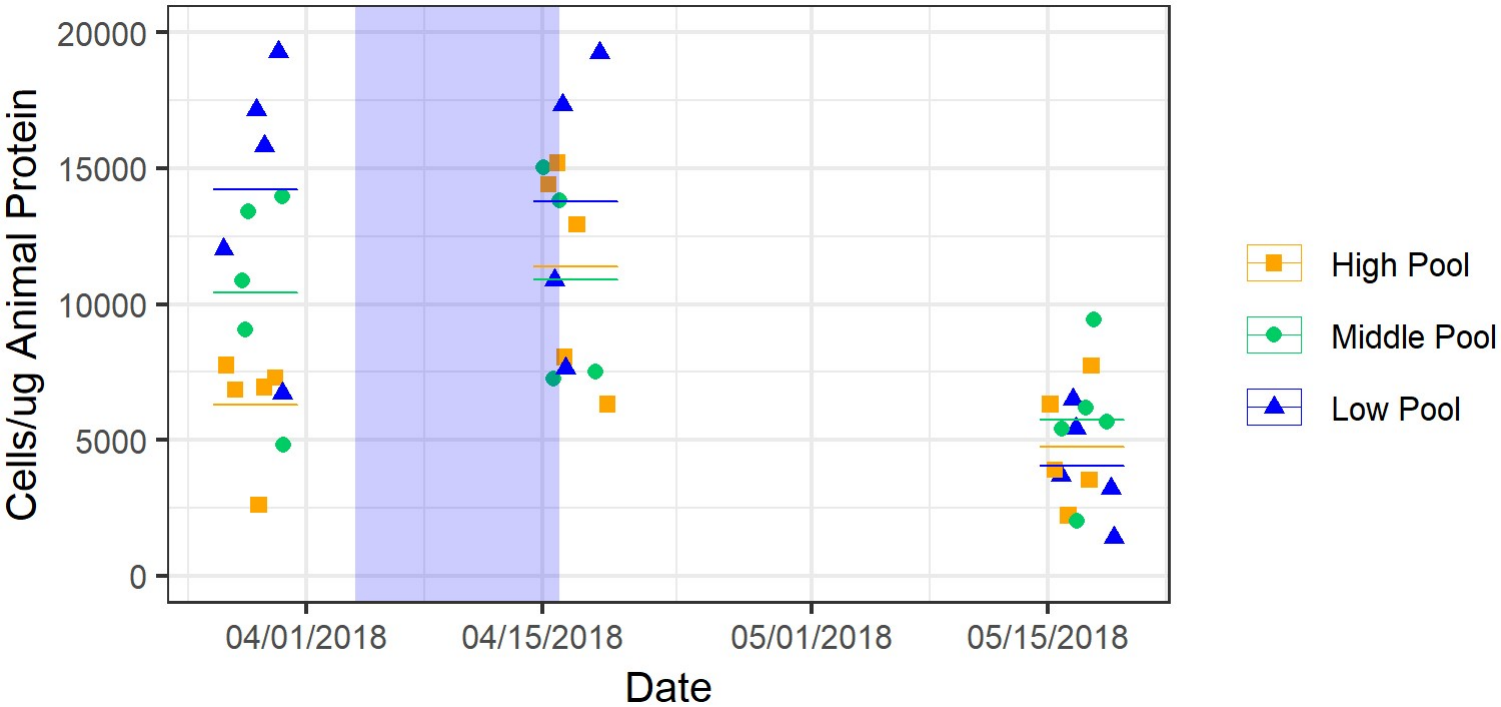
Symbiont abundance across tidal heights at Point Bonita. Bars show the mean for each colony sampled on each survey. Symbiont abundance is dependent on tidal height in the first survey (03/29/2018). Abundance measurements converge in the second survey (04/17/2018) and decrease by the third survey (05/17/2018). Mean symbiont abundance ± SD in High Pool was 6286 ±2084, 10994 ±4477, 4738 ±2236; Middle Pool was 10422 ±3712, 10894 ±4102, 5745 ±2638, and Low Pool was 14207 ±4955, 13775 ±5435, 4035 ±1970 over the three surveys. The blue shading represents the period of strongest freshwater influence as measured by the Fort Point buoy.

Particular focus is given to the tidepools at Point Bonita, the most proximal to the bay outflow. The relationship of tidal height and symbiont density at Point Bonita was dependent upon tidal height in the first survey (ANOVA, F = 10.33, p = .007) (Fig 5). In the second survey, symbiont density was no longer dependent upon tidal height (ANOVA, F = 0.9, p = .35). In the third survey density remained independent of tidal height (ANOVA, F = 0.5, p = .47). Between the second and third surveys, all colonies experienced more than a 50% decrease in mean symbiont density (Tukey HSD, p < .05 for all colonies) (Fig 5). All sampled colonies showed a decreased mean symbiont density to a value around 5000 cells/μg. This decrease is coincident with the documented decrease in salinity and minor increase in temperature (Fig 3). Five inches of rain was recorded near Point Bonita from 04/5/2018 to 04/7/2018. No other notable onsite precipitation was recorded during the survey period. Results of the field observations show a bleaching signal at the next sampling event close to two weeks after freshwater outflow events, suggesting that freshwater signals possibly impact intertidal habitats and longer controlled experiments may show a stronger signal to temperature and salinity stress. Slide Ranch and Rockaway tidepools, sites more distant from the outflow compared to Point Bonita, showed no significant change in symbiont abundance across sampling dates (Tukey, p>.1 for all pools).

### Coincident observation of burial and repopulation at Mile Rock

**Fig 6:**
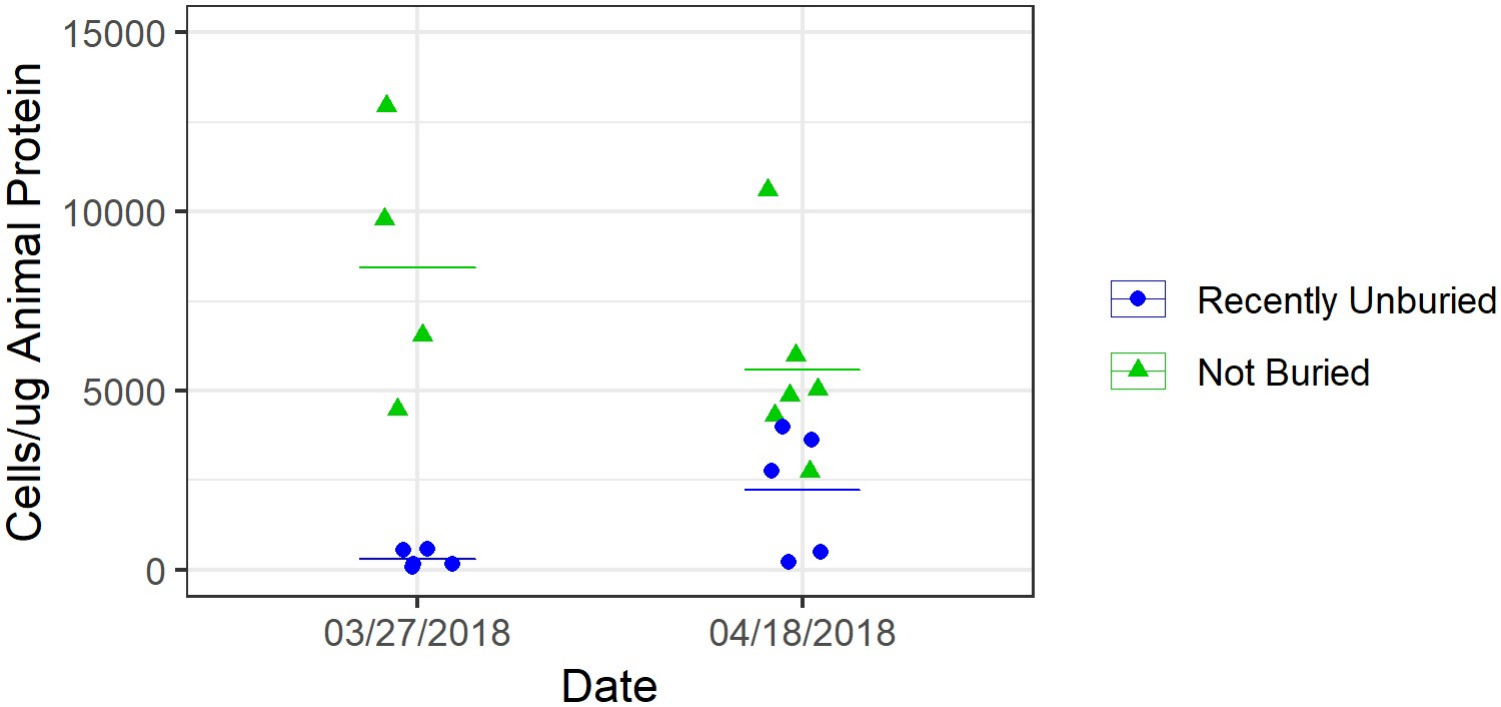
Symbiont abundance measurements after 9 months of natural burial. Symbiont repopulation in anemones was sampled after unburial of a single tidepool at Mile Rock. Bars show the mean for each colony sampled on each survey. The colony was sampled within days of unburial (03/27/2018), and again after three weeks (04/18/2018) along with a colony that was not buried. In the first survey, anemones in the previously buried pool exhibit near-zero numbers of symbionts. By the second survey, some anemones had comparable values to anemones in a colony that had not been buried. Mean symbiont abundance in the Recently Unburied pool went from 309 ± 242 to 2219 ±1767 cells/μg while the mean of the Not Buried pool went from 8434 ±3713 to 5591 ±2680 cells/μg over the survey period.

A sampled pool at Mile Rock filled with a single colony of *A*. *elegantissima* was buried for over 9 months due to natural sand fluctuation. Once unburied naturally, the anemones were found notably smaller and desiccated yet alive. Subsequent sampling of the same pool in the following weeks showed a statistically significant 700% increase in mean symbiont density (from 309 ± 242 cells/μg to 2219 ± 1767 cells/μg) in the colony (ANOVA, F = 5.7, p = .044). Fig 6 shows the repopulation of symbionts in the recently unburied anemones compared to a colony that was not buried.

### Controlled setting

**Fig 7:**
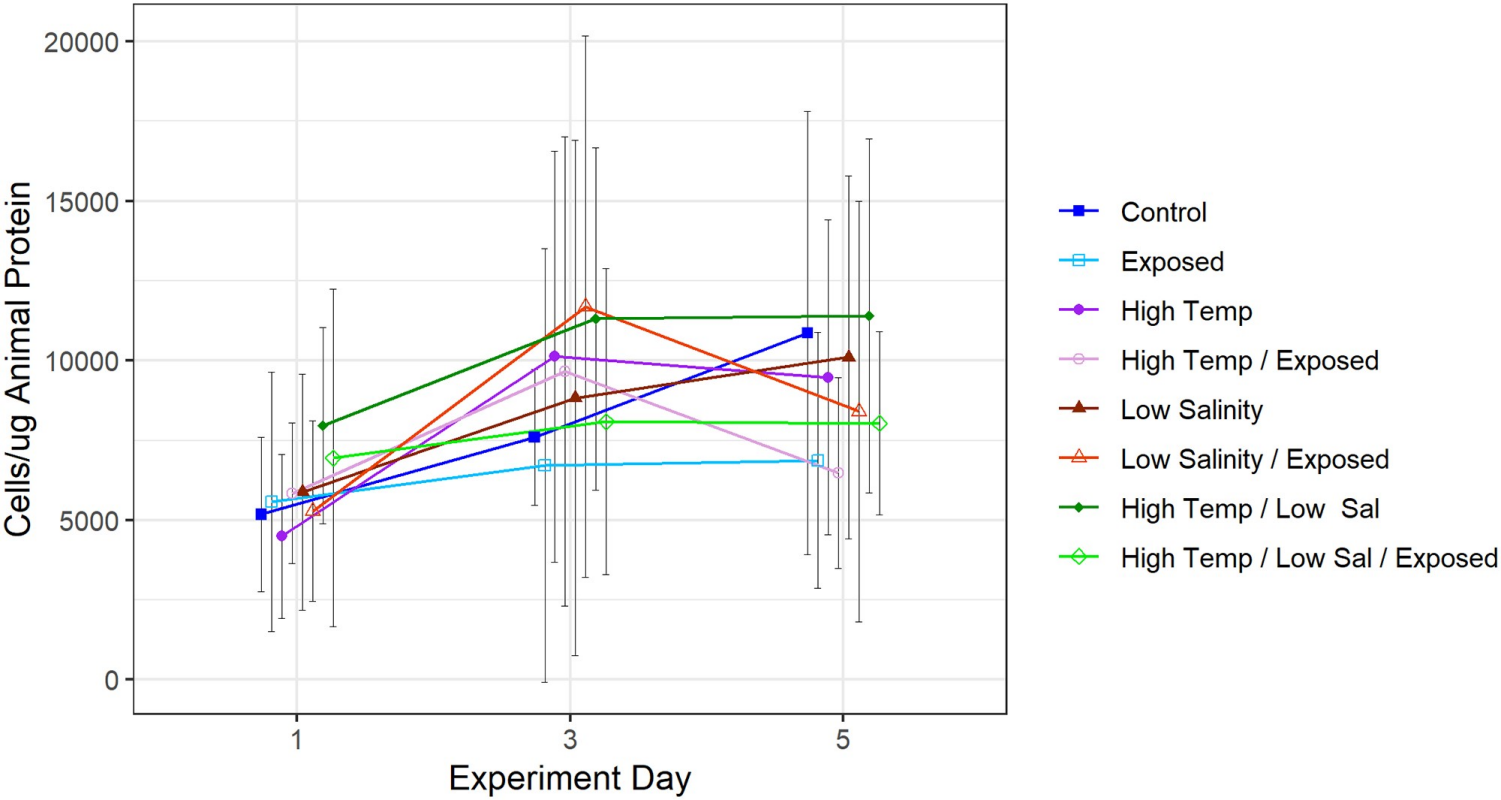
Pulsed 3-Stressor Experiment results. N = 5 per point. Error bars show ±CI. No treatment combination showed significant differences in symbiont abundance compared to other combinations. All treatment combinations appeared to increase slightly between Experiment days 1 and 3.

**Fig 8:**
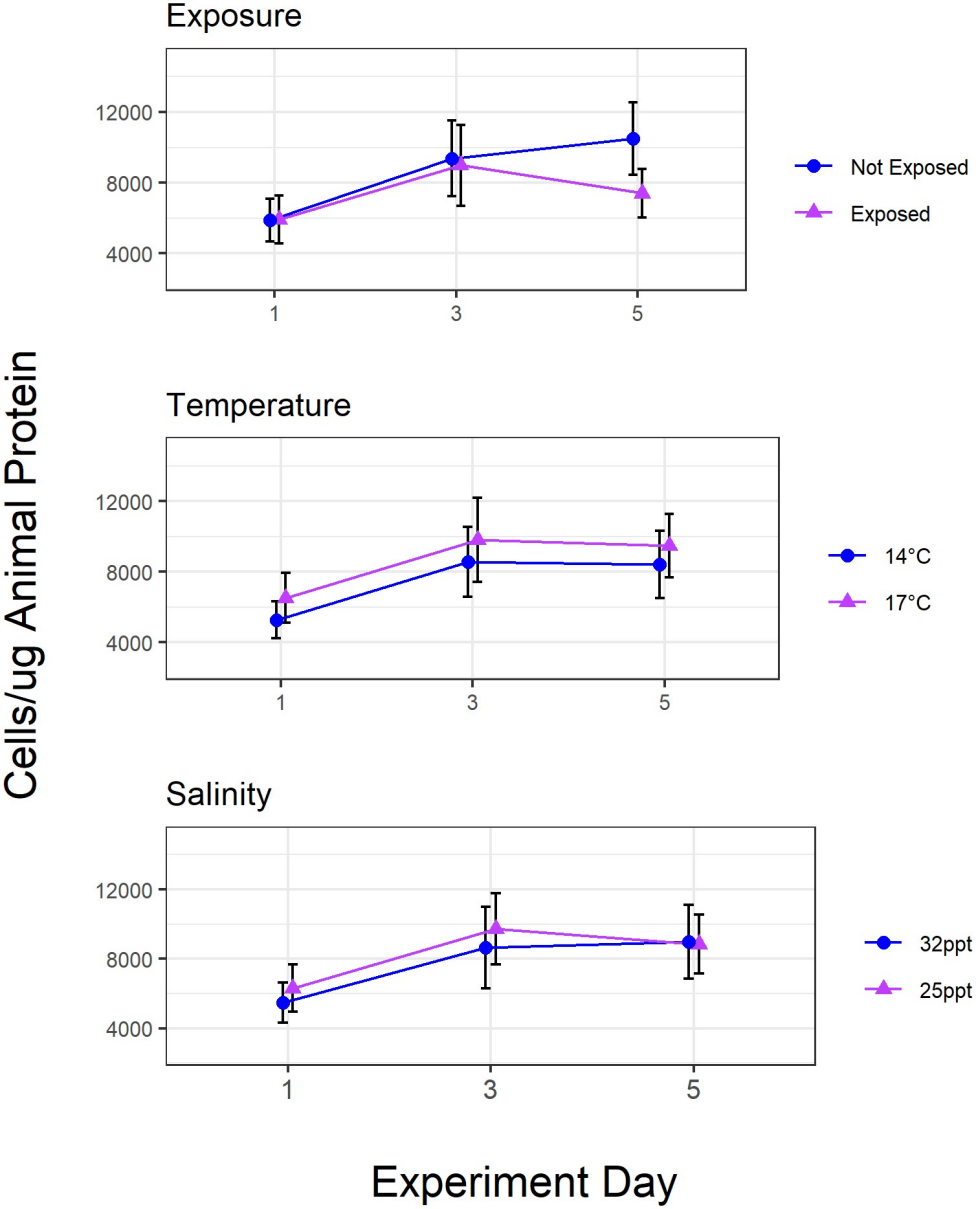
Treatment-specific results from the Pulsed 3-Stressor Experiment. N = 20 per point, error bars show ± CI. The aerial exposure treatment showed significant differences from the not exposed treatment by the end of the experiment. The salinity and temperature treatments did not show significant differences by the end of the experiment. Mean symbiont abundance in the Not Exposed treatment was 5881± 2576, 9371 ±4454, and 10503 ±4122 cells/μg compared to 5913 ±2872, 8986 ±4463, and 7397 ±2879 cells/μg in the Exposed Treatment over the three survey days through the five-day experiment.

Results from the Pulsed 3-Stressor Experiment show a statistically significant effect of mimicked low tide exposure on symbiont density populations (ANOVA, F = 6.3, p = .017) (Figs 7 and 8, Table 2). Aerial exposure had a large effect size (d = 0.87). No significant effect of altered temperature (17°C) and salinity (25ppt) was detected. The interaction between any two or three of the independent variables tested did not affect symbiont density during the course of the Pulsed 3-Stressor Experiment (ANOVA, p>.05 for all interactions).

**Table 2:** ANOVA results from the Pulsed 3-Stressor Experiment.

Treatment compared to Control	F value	p value
High Temperature	0.71	.404
Low Salinity	0.03	.860
Exposure	6.3	.017
High Temp *x* Low Salinity	0.24	.623
High Temp *x* Exposure	0.1	.75
Low Salinity *x* Exposure	0.007	.93
High Temp *x* Low Salinity *x* Exposure	0.27	.6

Lack of effect from single variable treatments may be the result of an underpowered experiment. Power analysis of the Pulsed 3-stressor experiment revealed a design with about 26% power. To achieve 80% power with a similarly large effect size as in the aerial exposure treatment, we would have needed at least 16 anemones per treatment. By undertaking a fully crossed multifactorial design, and despite being constrained by aquaria space, this portion of the study serves as a foundation for future statistically higher-powered experiments.

## Discussion

Results fit expectations of systematic bleaching events brought on by estuarine outflow and low tide series. The combination of field observations and controlled experiments suggest that the *A*. *elegantissima / B*. *muscatinei* symbiosis is susceptible to ongoing environmental variability, and will be affected by climate change. Field observations suggest a systematic bleaching event when low-salinity water parcels persist at intertidal sites. Anemones survived burial events lasting nine months, up to three times longer than previously recorded, and showing resilience to a common disturbance event [60]. Results from the controlled multifactorial experiment show the importance of aerial exposure over moderate changes to sea surface temperature and salinity, suggesting that increased aerial temperatures due to climate change [18,61] are likely to spur future bleaching events. The inclusion of tidal simulation in controlled experiments is recommended due to field and lab results.

### Temperature and freshwater input

The freshwater pulse documented at Fort Point for the wet spring season of 2018 was moderate compared to past years, but the timing presents an opportunity to compare plume presence to symbiont density at Point Bonita, the site most directly influenced by bay runoff. Symbiont density across the sampled tidepools decreased by 50% with persistent presence of the plume, and tidepools with symbiont density values that had previously been dependent on tidal height all decreased to a common “low” density, roughly 5000 cells/μg. The low density found for these tidepools suggests that the disturbance was enough to reset prior differences among local zones. The influence of tidal height on symbiont density is consistently shown in the results of the field observations, but the significant decrease in symbiont density due to freshwater input suggests the importance of these low salinity water parcels on the symbiosis. The timing of detectable effects could vary locally in comparison to salinity measurements at off-site buoys, which could encourage similar observations in a smaller, higher-resolution study area in future investigations. Freshwater input will continue to affect the symbiosis as climate change will increase the intensity of major freshwater runoff events.

### Field setting

The influence of tidal height on symbiont density is consistently shown in the results of the field observations. The surveyed tidepools ranged more than 1.5 meters of vertical height in the intertidal zone, demonstrating the hardiness of the *A*. *elegantissima / B*. *muscatinei* symbiosis. The results suggest that exposure and tidal height affect symbiont density as hypothesized, as tidepools at the higher vertical sampling limit had lower symbiont densities (colony means as low as 760 cells/μg protein) compared to equivalent pools at lower vertical sampling heights (colony mean as high as 14200 cells/μg protein). However, in comparing symbiont density to tidal height across all surveyed sites it is apparent that the highest pool (at +2m above MLLW) skews the grouped data towards linearity (Fig 4). This notable decrease in symbiont density above 2m could signal a tipping point above which *B*. *muscatinei* are unable to flourish. However, mean symbiont density decreases as tidal height increases at each individual site suggesting a possible dependent relationship (Fig 4). It is possible that tidal height affects symbiont density but site heterogeneity may decrease the signal.

### Coincident observation of burial and repopulation at Mile Rock

Previous studies have documented burial survival in *A*. *elegantissima* up to three months [49,62]. The incidental observation in this study shows the extreme hardiness and survival capabilities of this species, as colonies were buried for nine months under at least 0.5 meters of sand (at + 0.6m above MLLW). The anemones exhibited extreme desiccation upon unburial, but unburied individuals began near-immediate repopulation of their symbionts. Within three weeks, the unburied colony went from near-undetectable symbiont count to amounts comparable to many other colonies with “low” density, around 5000 symbionts per microgram of protein (Fig 6). This repopulation is either rapid horizontal integration or a repopulation by a residual symbiont population that was maintained even in prolonged total darkness as found in previous laboratory work with *Aiptasia* anemones [63].

Despite a similar symbiotic association with Symbiodiniaceae algae, there appears to be a stark contrast in resilience between these intertidal sea anemones and tropical reef-building corals. The incidental observation of *A*. *elegantissima* colonies that survived nine-month burial under sand in this study shows the extreme hardiness of the holobiont to the absence of light and heterotrophic feeding. In contrast, relatively narrow deviations in environmental factors (temperature, salinity, light, oxygen, N-to-P ratio) that underpin optimal metabolic functioning in corals can all result in bleaching [64]. For most coral species, bleaching without repopulation of endosymbionts or significant increase in heterotrophy leads to mortality [65]. An intriguing hypothesis to explain this contrast is that the physical environment has shaped the homeostasis of the cnidarian-Symbiodiniaceae symbiosis. Transplantation studies of corals in Ofu suggest that corals native to a Highly Variable Temperature pool have consistently higher levels of resistance to bleaching than those in a Moderately Variable Temperature pool [66]. *Anthopleura elegantissima* may be a more extreme case of the natural experiment on Ofu, where adaptation to wider environmental variation of the intertidal zone has armored the holobiont with greater resilience.

### Controlled setting

Experimental testing of temperature, salinity, and aerial exposure at amounts comparable to conditions near the San Francisco Bay confirms the importance of tidal height and aerial exposure over temperature and salinity when considering realistic predicted parameter ranges. Aerial exposure treatments induced a statistically significant stall of symbiont repopulation, while slightly elevated temperatures and moderate influence of freshwater based on modulation by changing tides did not affect symbiont density in the Pulsed 3-Stressor Experiment.

The controlled setting experiment suggests that moderately decreased salinity (25ppt) does not affect symbiont density within a five-day period in daily pulsed 3-hour exposures. This finding is consistent with previous work looking at a single stressor of constantly applied low salinity over a larger concentration range. Martin *et al* observed an 80% decrease of symbionts at a concentration of 25% saltwater (8ppt), a higher freshwater concentration than our treatments, within a five-day period [47]. Our moderate treatments were meant to reflect relatively frequent seasonal occurrences near San Francisco Bay. Alternatively, the lack of effect from low salinity may be due to the short experimental window since we detected reduced symbiont density at Point Bonita about two weeks after the freshwater plume.

### Significance

This study was designed to explore moderate temperature and salinity alteration, and to include for the first time an exposure treatment which more closely mimics current common field conditions. It is clear, from the significant differences in exposed and not exposed treatments in such a short time period, that including exposure considerations in experiments is crucial to evaluate current effects on this symbiosis. Additionally, the unanticipated documentation of anemone survival and rapid recolonization of symbionts after 9 months of burial is a novel finding and bolsters the case that *A*. *elegantissima* maintains a robust symbiosis with Symbiodiniaceae in contrast to many coral species.

Aerial temperature increase, sea surface temperature increase and changes in storm-induced salinity fluxes are predicted concerns for California coastlines as anthropogenically forced climate change progresses [18,20,22]. This study provides an example symbiotic Cnidarian system that is directly affected by these changes. As climate change progresses, systematic bleaching may become more prevalent and severe, causing further alterations to a sensitive food web. Exploring these interactions near major discharge sites provides insight for estuarine and coastal freshwater outflow.

There are no current studies outlining the effects of outflow plumes on *A*. *elegantissima* across its broad geographic distribution, although the species is found across the entire west coast of North America up into Alaska and is subject to freshwater input across the entire range. Anemone tentacle assays are a cheap method that provides indication of system health without lethal harvest and can be adapted for assessment across broad geographic regions. This study provides a quick, non-invasive method of investigating local intertidal primary productivity. The study of this ecosystem health indicator could prove useful for accessible baseline ecosystem monitoring.

### Future directions

Future studies may include examining genetic linkages to stress resistance and symbiont relationships in *A*. *elegantissima*, as found in certain coral species [67–69], especially with respect to differential exposure [70]. Modeling *B*. *muscatinei* food web contribution in various climate scenarios could predict intertidal productivity changes [71]. Further questions related to heterogeneous salinity tolerance within *A*. *elegantissima* colonies can be addressed by testing whether inner polyps are buffered from freshwater influence by outer polyps. Due to the observational nature of the field portion of this study, further investigation into the effects of the seasonal large-scale ecological disturbance on local *A*. *elegantissima / B*. *muscatinei* symbiosis is warranted. A longer-term controlled setting experiment in tandem with long term monitoring of the symbiosis could shed light on the question of response times raised in this study.
